# 抗肿瘤药物警戒研究进展

**DOI:** 10.3779/j.issn.1009-3419.2022.101.33

**Published:** 2022-07-20

**Authors:** 雯娟 孙, 扬 胡, 燕 徐, 波 张

**Affiliations:** 1 100730 北京，中国医学科学院，北京协和医学院，北京协和医院，药剂科 Department of Pharmacy, Peking Union Medical College Hospital, Chinese Academy of Medical Sciences and Peking Union Medical College, Beijing 100730, China; 2 100730 北京，中国医学科学院，北京协和医学院，北京协和医院，呼吸与危重症医学科 Department of Respiratory and Critical Care Medicine, Peking Union Medical College Hospital, Chinese Academy of Medical Sciences and Peking Union Medical College, Beijing 100730, China

**Keywords:** 抗肿瘤药物, 药物警戒, 监测模式, 目标, Antitumor drugs, Pharmacovigilance, Monitoring mode, Target

## Abstract

2019年我国新修订的《药品管理法》首次提出国家建立药物警戒制度，2021年我国首部《药物警戒质量管理规范》发布。药物警戒的提出和实施在我国为初始阶段，从体制、监测机制、数据库建设等方面需不断完善。我国肿瘤患者新发病例数量位居全球首位，近年来，新型抗肿瘤药物上市速度加快，临床试验繁多，抗肿瘤药物警戒工作势在必行。本文从药物警戒起源、临床实践目标、流程与方法、抗肿瘤药物警戒难点及我国药物警戒现行特点等方面归纳总结，旨在为抗肿瘤药物警戒的开展提供参考。

## Abstract (en)

In 2019, Drug Administration Law of China was first time proposed that the country should establish pharmacovigilance system. In 2021, the first Pharmacovigilance Quality Management Standard of China was released. The proposal and implementation of pharmacovigilance were the initial stage in China, and it needed to improve the aspects of pharmacovigilance include institution, monitoring mechanism and database construction. The number of new diagnosed cancer patients in China ranked first in the world. In recent years, the marketing speed of novel antitumor drugs was accelerated, and there were many clinical trials. Therefore, antitumor pharmacovigilance was imperative. In this article, we summarized pharmacovigilance of the origin, clinical practice objectives, procedures, methods. We described the difficulties in antitumor pharmacovigilance and current characteristics of pharmacovigilance in China, aiming to provide reference for the development of antitumor pharmacovigilance.

## 1 药物警戒的起源

20世纪60年代，“反应停事件”引发全球对于药品安全问题的关注。此后，世界卫生组织（World Health Organization, WHO）强调加快不良反应信息传递和及早采取行动的重要性，并设立国际药物监测试点研究项目，各国纷纷设立药品不良反应报告系统。WHO的药品不良反应（adverse drug reaction, ADR）定义为：药品在正常用法用量下出现的非预期的有害反应。2002年，WHO将药物警戒（pharmacovigilance, PV）定义为“与检测、评估、理解和预防不良反应或任何其他药品相关问题有关的科学研究和活动”^[1]^。2015年，WHO发布的《药物警戒系统实用评估手册》^[2]^指出：药物警戒不仅涉及不良反应和不良事件监测，还包括用药差错、假药或劣药、药物无效、药物误用和（或）滥用、药物相互作用等。药物警戒涵盖的范围除常规药品外，还包括草药、传统和补充产品、生物制品、疫苗、血液制品和可能涉及的医疗设备等。药物警戒制度在原有ADR监测和报告制度基础上进行了延展，体现在药品上市后管理和临床试验阶段的动态风险控制，包括疑似ADR的监测和报告制度、药品风险管理计划制度、处理药品质量问题或其他与药品相关的安全隐患制度等^[3]^。

## 2 药物警戒临床实践目标

①改善患者用药护理和用药安全性，改善医疗和辅助医疗干预措施；②改善公共卫生和用药安全；③协助评估药物的效益、危害、有效性和安全性，鼓励安全、有效、合理用药（包括成本效益）；④促进对药物警戒的理解、教育和临床培训，促进与卫生专业人员和公众进行有效沟通。

## 3 药物警戒的参与者

政府、企业、医院、学术界、医药协会、不良反应监测机构、卫生专业人员、患者、媒体、WHO等。

## 4 医院和临床实践者的责任和义务

医疗机构开展的药物警戒内容包括药物警戒信息系统建设、病历数据监测、个体化血药浓度监测等。常用药品的安全监测是药物警戒临床实践的重要组成部分。临床医生对药物警戒的了解程度、执行程度对卫生保健的质量影响很大。加强卫生专业人员的药物安全教育与培训，与国家ADR监测中心进行有效交流，并将用药安全的临床经验与研究同卫生政策链接，有助于加强患者照护。

## 5 药物警戒方法与流程

WHO将药物警戒方法分为被动监测（如自发报告）、主动监测（哨点机构监测、处方数据监测、疾病或药物登记研究）、刺激报告以及对比观察性研究（横断面调查、病例对照研究、队列研究）等^[4]^。国家层面开展的药物警戒以自发报告为基础。通过自发报告系统收集、整理和分析疑似不良反应报告，便于信号检测和风险管理。在地方一级，医务工作者、药品上市许可持有人（marketing authorization holder, MAH）、患者等将疑似不良反应报告提交到区域或国家中心。全球范围内的个例安全性报告（individual case safety report, ICSR）数据库名为Vigibase，由WHO乌普萨拉监测中心（Uppsala Monitoring Centre, UMC）维护，UMC基于VigiBase的数据开展信号检测，并及时通报给各国^[3]^。自发报告系统是上市后药品不良反应的主要报告渠道，报告数量巨大，但存在漏报等局限性^[5, 6]^。

欧盟自发报告流程即不良反应报告首先以在线或纸质报告方式发送给成员国物警戒联系人或MAH，审核并确认有效的ICSR及时上报欧盟药物警戒数据库（EudraVigilance, EV）。如意大利自发报告不良反应流程，不良反应报告首先经药物警戒联系人审核后，上报意大利药品管理局（Agenzia Italiana del Farmaco, AIFA）的药物警戒网络（Rete Nazionale di Farmacovigilanza, RNF）及区域药物警戒系统，RNF与EV相关联；MAH接收的ADR可直接报告EV，EV将自发报告上传UMC并反馈给RNF和MAH。在自发报告基础上的信号检测，是确定新发ADR或已知ADR频率变化的第一步。欧洲药品监督管理局（European Medicines Agency, EMA）将信号定义为来自一个或多个来源的信息，预示新的潜在因果联系并可以证明采取核查行动合理性的信号。EMA药物警戒质量管理规范^[7]^指出，信号管理过程包括EMA和MAH的检测、验证、确认，之后由EMA药物警戒风险评估委员会（Pharmacovigilance Risk Assessment Committee, PRAC）分析、优化、评估信号，必要时采取监管行动。MAH接收信号检测产生的新信息用来修改风险管理计划^[8, 9]^。2012年，欧洲药物警戒立法引入对特定药物进行额外监测的新措施即由黑色倒三角形代表的额外监测药物（Medicines Under Additional Monitoring, MUAM）^[10]^，目的在于鼓励卫生专业人员和消费者报告可疑的ADR，有效促进药品获益-风险分析。2013年-2017年MUAM清单分析结果^[11]^显示，清单内66%药品含有新活性物质，主要为抗肿瘤药物和免疫调节药物；其中，酪氨酸激酶抑制剂出现频率较高。MUAM清单中1/3为生物药物，包括蛋白质类、人体代谢酶、单克隆抗体、血液制品以及免疫药物、基因和细胞产品等。基于此类药品的化学特性和安全特性，药物警戒对于额外监测药物更有意义。MUAM包括：①EMA批准的含有新活性物质的药品；②EMA批准的生物药品包括生物仿制药；③MAH需进行批准后安全性研究的药物；④有条件批准或特殊情况下批准的药品以及对可疑不良反应的记录或监测负有特定义务批准的药品。目前，MUAM目录内的药物额外监测期限为上市后5年或直到PRAC评估可从MUAM清单中撤销。

目前全球没有统一的主动监测模式，集中监测计划、处方事件监测、注册登记研究是接受性较高的方式^[12]^。美国食品药品监督管理局（Food and Drug Administration, FDA）建立了主动监测系统即哨点监测系统（the Sentinel Initiative），通过收集各医疗机构电子数据进行药物监测，由FDA分析并通过MedWatch计划和FDA网站向公众传播^[13]^。2003年，英国成立的Health Tracker是基于网络多模式的健康监测平台^[14]^，其目标即利用患者监测报告为临床医生提供用药适应证、副作用、满意度、生活质量等相关信息。

药物警戒体系的建立和实施可以提高肿瘤患者的生活质量，整合临床试验和研究的ADR报告数据在指南、工具和平台建设方面有重要意义^[15, 16]^。FDA不良事件报告系统（FDA Adverse Event Reporting System, FAERS）是美国药物警戒数据库^[17]^，对所有上市药品和治疗用生物制品的安全性进行监测，包括ADR报告、用药错误报告和产品质量问题等类型的所有报告，药品评价与研究中心和生物制品评价与研究中心筛选FAERS的安全性信息并检测安全信号，开展科学的临床评估，并根据评估结果提出监管建议（如建议修改标签、改变销售授权或确认是否采取进一步行动等）。日本药物警戒数据库（Japanese Adverse Drug Event Report, JADER）收集的报告由日本药品和医疗器械管理局（Pharmaceuticals and Medical Devices Agency, PMDA）审查部门评估报告、筛选安全信号、与制药企业交换信息、组织专家委员会对重点案例进行讨论等，并向日本厚生劳动省（Ministry of Health, Labour, and Welfare, MHLW）提出行动建议，由MHLW负责采取适当的措施。评估过程中亟需采取监管措施的，可以通过《医务人员紧急安全性信息告知函》的形式发布安全提示；重要但不紧急的安全性问题可通过《医务人员安全性信息快速告知函》的形式发布。EV目前已拥有1, 860多万例ICSRs，是世界上最大的药物警戒数据库之一^[18]^。EV数据主要来源于欧盟各成员国药监部门、制药企业和临床试验申办者。欧盟成员国药品监管局、药物警戒风险评估委员会和EMA共享数据库信息，定期审查和分析EV数据以检测药品安全信号。此外，EMA与许多国家和组织建立合作，并向WHO通报集中授权的药品采取的可能对欧盟以外国家的公共健康保护有影响的措施。

## 6 抗肿瘤药物警戒难点

抗肿瘤药物警戒的8个关键问题：不良反应术语；不良反应范围；靶向治疗和免疫治疗；化疗；仿制药和生物类似物；药物相互作用、药物基因组学和多重用药；特殊人群；不良反应少报或漏报。

抗肿瘤药物不良反应是肿瘤患者治疗期间面临的最重要问题之一。传统抗肿瘤药物安全性特点包括固有毒性、治疗窗窄、高剂量和间歇给药治疗方案等。近10年，全球抗肿瘤药物的研发数量和速度加快，新型抗肿瘤药物不断涌现。2018年，美国44%的转移性实体或血液肿瘤患者使用免疫检查点抑制剂（immune checkpoint inhibitors, ICIs）。肿瘤治疗方案除传统化疗外，还包括靶向药物治疗、ICIs单药或联合治疗模式（例如ICIs与化疗或靶向药物联合）等^[19]^，ICIs覆盖肿瘤早期到晚期的整个疾病谱系，肿瘤治疗方案从传统化疗药物发展为多药联合方案，抗肿瘤药物警戒管理难度和复杂性显著提高。

### 6.1 化疗药物、新型抗肿瘤药物的不良反应异同

新型抗肿瘤药物是指小分子靶向药物和大分子单克隆抗体类药物。其中，ICIs与传统细胞毒类药物的药效学和药代动力学差异较大，毒谱分布有所不同。传统细胞毒类药物引发的急性发作呕吐和骨髓抑制等更加常见，而免疫相关不良反应（immune relate adverse effects, irAEs）往往是发作延迟、炎性或自身免疫性质的不良反应。另外，早发和迟发的irAEs引发机制可能不同。典型的早发、常见的irAEs表现为广泛性上皮炎症，包括皮疹、结肠炎和肺炎等；迟发性irAEs通常不太常见，包括神经系统事件和垂体炎等，这些反应往往是局部的、器官特异性反应。单药ICIs治疗相关的任何级别irAEs的发生率为15%-90%，严重irAEs发生率为0.5%-13%^[20]^。免疫联合治疗时，43%的患者因iAEs而停止联合治疗（纳武利尤单抗/伊匹木单抗）。其中，胃肠道不良事件是最常见的停药原因^[21]^。

### 6.2 肿瘤治疗方案复杂

目前，肿瘤治疗方案包括细胞毒药物、内分泌药物、小分子靶向药物和大分子单抗类药物等，药品种类繁多。除此之外，肿瘤治疗还涉及预防和处理不良反应的预处理药物、镇痛药物、止吐药物以及基础病的慢病用药等多药联用的情况，均可能增加药物相互作用风险及疗效差异^[22]^，并给不良反应的关联性评价带来困难。

### 6.3 特殊人群

在肿瘤治疗中，孕妇、老年和儿童人群需要特殊关注。孕期合并肿瘤的现象并不罕见，例如乳腺癌是最常见的伴发肿瘤，其次是黑色素瘤和血液系统肿瘤等^[23]^。此类患者应用全身化疗的临床经验较少，对胎儿发育和生长的长期安全性并不明确。同时需要选择对患者的健康和生活质量影响较小的药物。老年患者随着年龄增长而发生的生理变化（如肾衰竭、心血管损伤、代谢问题等）通常需要调整药物剂量和治疗方案。另外，老年患者存在用药依从性问题、居家服用靶向药物的安全性问题。药品说明书适应证中，儿童与成人差别较大，在肿瘤治疗中更为显著。因此，儿童肿瘤患者超说明书用药情况较多，用药安全性监管难度提高。

### 6.4 不良反应报告不全

不良反应少报或漏报现象常见。在英国，仅有10%的严重不良反应和2.4%非严重ADR被上报^[24]^。抗肿瘤药物出现如上现象的原因可能包括抗肿瘤药物的不良反应发生率高且难以避免，通常被认为是正常现象而不进行报告；对药物警戒态度和认知水平有待提高；对应报告的ADR类型（新的和严重的或全部）缺乏了解；医务人员工作繁忙；报告责任不明确；医院管理部门缺少反馈意见等^[18, 24, 25]^。因此，亟需相应改进措施提升药物警戒管理效果，如设置卫生专业人员，建立患者协作群或电子工具（例如APP、ADR网络上报平台），开展ADR安全信号的早期监测，设立药物警戒数据库等。

## 7 我国药物警戒现状

2019年12月，我国新修订的《药品管理法》正式实施，第一次提出国家建立药物警戒制度，即“对药品不良反应及其他与用药有关的有害反应进行监测、识别、评估和控制”。2021年5月，我国首部《药物警戒质量管理规范》发布。与此前的ADR监测相比，药物警戒涵盖药品整个生命周期的全方位的安全监管，除了关注狭义上的ADR外，还关注药品滥用、误用、药物相互作用等其他与用药有关的安全问题；同时药物警戒的时间覆盖范围也更广，贯穿于药品上市前研究、上市后安全性监测及评价直至最后的撤市或淘汰的整个药品生命周期。我国目前需坚持“一体两翼”的不良反应监测格局，另外需加强药品上市前审批、上市后监测评价等部门的有效沟通协调机制，针对药品误用、错用、不合理使用等引发医疗风险，但尚不属于ADR监测系统主动收集范围的内容，需设立有效的监测和管控方法，不断完善药物警戒体系。

## 8 结语

药物警戒在我国为初始阶段，从定义和内涵、管理制度体系、组织体系、技术规范体系、考核评估机制以及社会共治机制等都需不断完善；近年来，肿瘤患者数量攀升，新型抗肿瘤药物上市速度加快，临床试验数量繁多，抗肿瘤药物警戒工作势在必行，应加强抗肿瘤药物警戒监测机制、数据库建设、信号挖掘等工作，保障肿瘤患者用药安全。
